# The YcnI protein from *Bacillus subtilis* contains a copper-binding domain

**DOI:** 10.1016/j.jbc.2021.101078

**Published:** 2021-08-14

**Authors:** Madhura S. Damle, Aarshi N. Singh, Stephen C. Peters, Veronika A. Szalai, Oriana S. Fisher

**Affiliations:** 1 Department of Chemistry, Lehigh University, Bethlehem, Pennsylvania, USA; 2 Department of Earth and Environmental Sciences, Lehigh University, Bethlehem, Pennsylvania, USA; 3 Physical Measurement Laboratory, National Institute of Standards and Technology, Gaithersburg, Maryland, USA

**Keywords:** metal ion–protein interaction, metalloprotein, bacteria, crystallography, electron paramagnetic resonance, DUF1775, Domain of Unknown Function 1775, EPR, electron paramagnetic resonance, HMM, hidden Markov model, ICP-MS, inductively coupled plasma-MS, ITC, isothermal titration calorimetry, JGI-IMG, Joint Genome Institute-Integrated Microbial Genomes, LPMO, lytic polysaccharide monooxygenase, Ni–NTA, nickel–nitrilotriacetic acid, pCuAC, periplasmic copper A chaperone

## Abstract

Bacteria require a precise balance of copper ions to ensure that essential cuproproteins are fully metalated while also avoiding copper-induced toxicity. The Gram-positive bacterium *Bacillus subtilis* maintains appropriate copper homeostasis in part through the *ycn* operon. The *ycn* operon comprises genes encoding three proteins: the putative copper importer YcnJ, the copper-dependent transcriptional repressor YcnK, and the uncharacterized Domain of Unknown Function 1775 (DUF1775) containing YcnI. DUF1775 domains are found across bacterial phylogeny, and bioinformatics analyses indicate that they frequently neighbor domains implicated in copper homeostasis and transport. Here, we investigated whether YcnI can interact with copper and, using electron paramagnetic resonance and inductively coupled plasma-MS, found that this protein can bind a single Cu(II) ion. We determine the structure of both the apo and copper-bound forms of the protein by X-ray crystallography, uncovering a copper-binding site featuring a unique monohistidine brace ligand set that is highly conserved among DUF1775 domains. These data suggest a possible role for YcnI as a copper chaperone and that DUF1775 domains in other bacterial species may also function in copper homeostasis.

Copper is an essential cofactor for many enzymes, but in high quantities, it can also have deleterious effects on cellular viability because of formation of reactive oxygen species. In nearly all bacterial species, a suite of proteins maintains an appropriate balance of copper by regulating its homeostasis and mediating its transport in and out of the cell (1, 2). Copper efflux is usually carried out by Cu(I)-exporting P-type ATPases, which often are further assisted in this process by additional proteins. For example, in the Gram-positive *Bacillus subtilis*, the CopA Cu-dependent ATPase exports copper with the aid of the CopZ chaperone (3, 4, 5). The *copZA* operon is further regulated by CsoR, a copper(I)-sensing repressor, that binds to the operator region in the absence of copper (6, 7).

Bacterial copper acquisition, on the other hand, has remained more elusive. Data from a number of different organisms, however, have converged to suggest that the CopD domain functions as a membrane-bound copper importer. Proteins containing CopD domains have been found to play an essential role in conferring copper resistance in a wide range of microorganisms including *B. subtilis* (8, 9), *Pseudomonas syringae* (10, 11), *Acinetobacter baumannii* (12), and *Bradyrhizobium japonicum* (13). These and other microbiological and transcriptomic studies strongly point toward a role for this domain in copper uptake (8, 9, 12, 14, 15, 16). Many proteins with CopD domains, however, are encoded by operons that include additional genes whose protein products have also been implicated in copper homeostasis such as *copC* and periplasmic copper A chaperone (*pCu*_*A*_*C*) (17, 18), and others whose protein products have yet to be functionally characterized.

One notable example is the *ycnKJI* operon in *B. subtilis* that encodes the YcnK, YcnJ, and YcnI proteins (Fig. 1*A*). YcnK is a Cu-dependent transcriptional repressor that uses a helix–turn–helix domain to bind to an intergenic region upstream of the *ycn* operon (9), controlling its expression. Because loss of *ycnJ* results in a reduction of intracellular copper levels (8, 9), the YcnJ protein has been proposed to serve as a copper importer. As further evidence for such a role, YcnJ is a fusion protein comprised of an extracellular CopC domain of the C_0–1_ type that typically binds Cu(II), a membrane-bound CopD domain, and a C-terminal YtkA domain of unknown function (18) (Fig. 1*B*).

**Figure 1:**
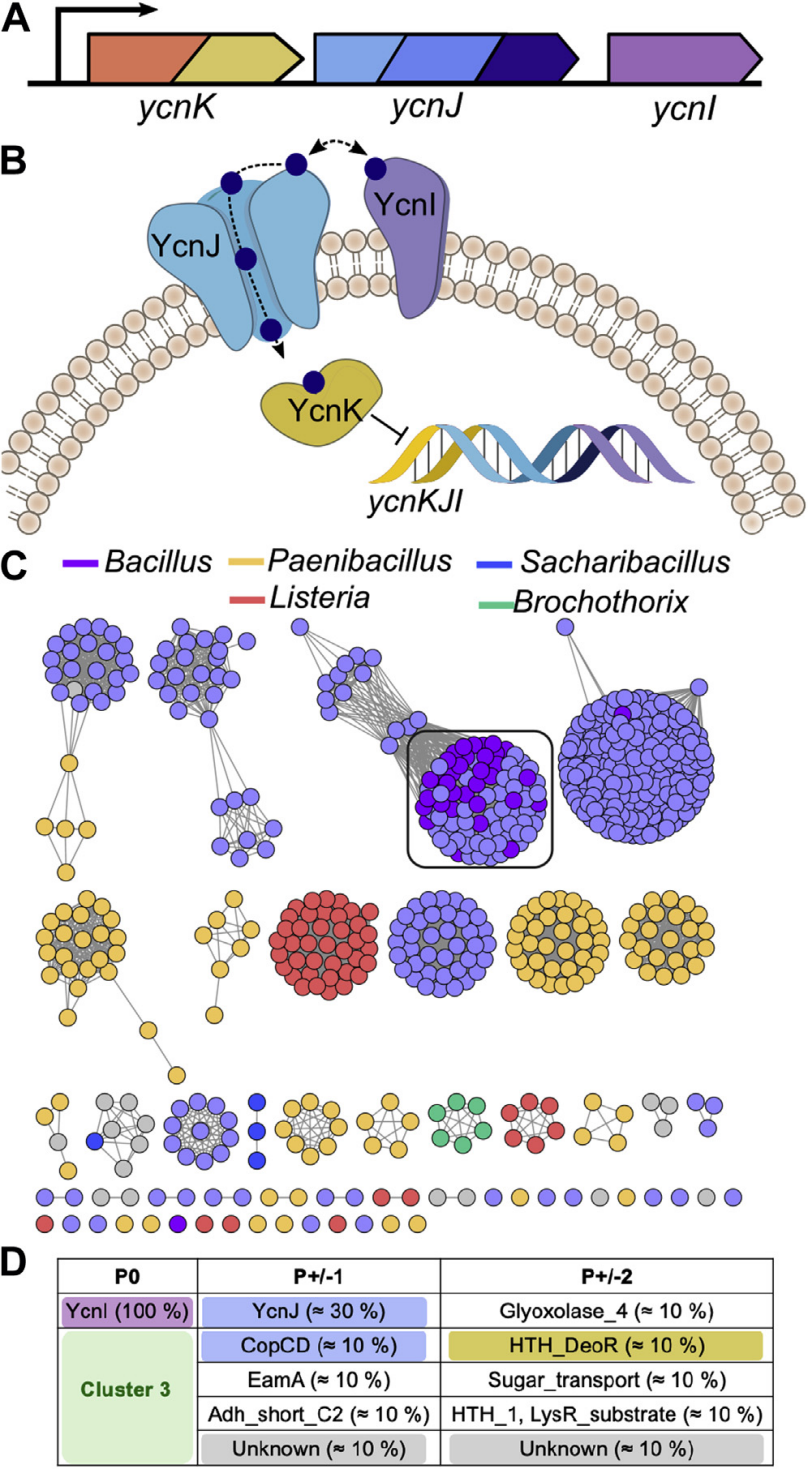
**Bioinformatics analysis of DUF1775 sequences most closely related to *Bacillus subtilis* YcnI.***A*, architecture of the *ycn* operon. *B*, schematic diagram showing the proteins encoded by the *ycn* operon and their proposed interactions with Cu (shown as *blue spheres*). *C*, sequence similarity network for DUF1775 sequences in the same cluster as YcnI colored by genus, alignment score cutoff of 70. Sequences are colored by taxonomy, and the cluster containing the *B. subtilis* YcnI is boxed. *D*, genome neighbors within two positions of the sequences shown in *A*. DUF1775, Domain of Unknown Function 1775.

The third protein encoded by the *ycn* operon, YcnI, remains much more poorly understood. It includes a Domain of Unknown Function 1775 (DUF1775) and a C-terminal transmembrane helix (8, 19). Its biological function has not been determined, but its frequent association with CopC and CopD suggests that it likely is also involved in copper homeostasis (9, 13, 17, 18, 20). Furthermore, YcnI is homologous to the PcuD protein encoded by the *pcuABCDE* operon from *Bra. japonicum* that also appears to play a role in copper acquisition (13). Studies of the *ycn* operon have alluded to a potential role for YcnI in copper homeostasis (9). The lack of biochemical information about YcnI and the absence of a recognizable metal-binding motif in its amino acid sequence, however, have impeded further advances toward elucidating its functional role.

Here, we investigate a link between YcnI and copper. We find that YcnI homologs are present in a wide range of bacterial species, with particularly high representation within the firmicutes, actinobacteria, and proteobacteria phyla, and that genes encoding DUF1775 domains frequently occur in close proximity to other copper-related genes. We recombinantly produce the soluble domain of *B. subtilis* YcnI *in vitro* and demonstrate that it binds Cu(II) in a 1:1 stoichiometry. To investigate the structure of the protein and to further probe the coordination geometry of the copper site, we determine crystal structures of the protein in both the apobound and Cu-bound states. The latter reveals an unusual copper-binding motif that we term the monohistidine brace because of its similarities to the canonical histidine brace coordination site used by some monooxygenases and other copper-binding proteins (17, 21, 22). The metal-binding ligands identified in the crystal structure are strictly conserved in the majority of YcnI sequences, suggesting a role for YcnI homologs in copper homeostasis and trafficking in many bacterial species.

## Results

### Genomic analyses suggest an association between DUF1775 domains and copper-binding proteins

To investigate the role of the DUF1775 domain in bacteria, we conducted a large-scale bioinformatics analysis of DUF1775 domains. We mined the Joint Genome Institute-Integrated Microbial Genomes (JGI-IMG) database (23) for sequences that match the associated protein family (PFAM), pfam07987, identifying over 10,000 individual sequences that we used to construct a sequence similarity network. Notably, DUF1775 domains appear throughout bacterial phylogeny, occurring in 9354 unique genomes and within both Gram-positive and Gram-negative organisms (Fig. S1*A*). The domain is most highly represented in the proteobacteria, actinobacteria, and firmicutes phyla, as well as a significantly smaller representation in deinococci. The majority (≈90%) of these sequences contain a single DUF1775 domain, whereas a subset of the sequences from proteobacterial species fuse the DUF1775 domain to a PCuAC domain (Fig. S1*B*). This corroborates previous studies of *DUF1775* genes that noted a co-occurrence with *pCu*_*A*_*C* and other genes such as *copC* and *copD* that encode copper-related proteins (9, 13, 17, 18).

To understand more about how the *B. subtilis* YcnI protein compares to other proteins with DUF1775 domains, we narrowed in on the cluster of DUF1775 sequences in which *B. subtilis* YcnI is found. *Bs*YcnI appears most closely related to DUF1775 domains found in other members of the *Bacillus* genus, as well as other firmicutes, most notably *Listeria* and *Paenibacillus* (Fig. 1*C*). Of these proteins, ≈40% (270 of a total 606 sequences) neighbor a CopCD-encoding gene, including those like *ycnJ* (Fig. 1*D*). Approximately 20% of these genes also are near a YcnK-like or helix-turn-helix-containing protein, comprising what, in *B. subtilis*, has been termed the *ycn* operon (Fig. 1*A*). Despite its frequent proximity to copper-associated domains, however, YcnI does not contain any known conserved metal-binding motifs. Examination of the sequence logo for the DUF1775 family reveals one very highly conserved histidine residue (present in 10,393 of 10,647 sequences) near the N terminus but no other obvious conserved residues or motifs for metal binding.

### *Bs*YcnIΔC binds Cu(II)

Because our bioinformatics data suggested a link between YcnI and proteins such as CopC, CopD, and PCu_A_C involved in copper homeostasis, regulation, and trafficking, we next sought to determine if YcnI binds copper. The *B. subtilis* YcnI protein contains a predicted signal sequence N-terminal to the DUF1775 domain, suggesting that endogenously, the DUF1775 domain is localized extracellularly and tethered to the membrane by the C-terminal transmembrane helix (Fig. S1*B*). Secondary structure predictions and sequence alignments suggested that upon cleavage of the signal peptide, the N-terminal residue would be the well-conserved histidine. We hypothesized that this histidine might serve a role as a metal ligand, analogous to how an N-terminal histidine residue coordinates copper ions in other bacterial proteins including lytic polysaccharide monooxygenases (LPMOs) (21, 24, 25, 26, 27, 28, 29), CopC (18, 22, 30, 31, 32), the PCu_A_C domain of PmoF (17), and the Cu_B_ site of particulate methane monooxygenase (33).

To generate the YcnI protein with the native N-terminal histidine residue intact, we engineered a small ubiquitin-like modifier (SUMO)–tagged construct of the DUF1775 domain of YcnI (*Bs*YcnIΔC). We expressed this construct in *Escherichia coli* and purified it to homogeneity after cleavage of the SUMO tag. To investigate the possibility that *Bs*YcnIΔC could bind copper, we incubated the purified protein with different stoichiometric ratios of CuSO_4_ and measured the concentration of bound copper by inductively coupled plasma-MS (ICP-MS) after removing unbound metal using a desalting column. We find that addition of either one equivalent or two equivalents resulted in 0.98 ± 0.09 Cu ions bound per protein, indicating that the protein binds the metal ion in a 1:1 stoichiometry when either one or two equivalents of the metal are added. The as-isolated apo samples did not have significant copper content (0.081 ± 0.06 Cu ions per protein monomer) (Table 1).

**Table 1:** YcnIΔC copper-binding stoichiometry

Sample	Mole equivalent Cu:protein monomer
*Bs*-YcnIΔC, apo	0.08 ± 0.06
*Bs*-YcnIΔC, Cu-loaded^a^	0.98 ± 0.09

a Samples were incubated in a one to one ratio with CuSO_4_ prior to desalting. Uncertainties are standard deviations, calculated from three (apo) and five (Cu-loaded) independent replicates.

**Figure 2:**
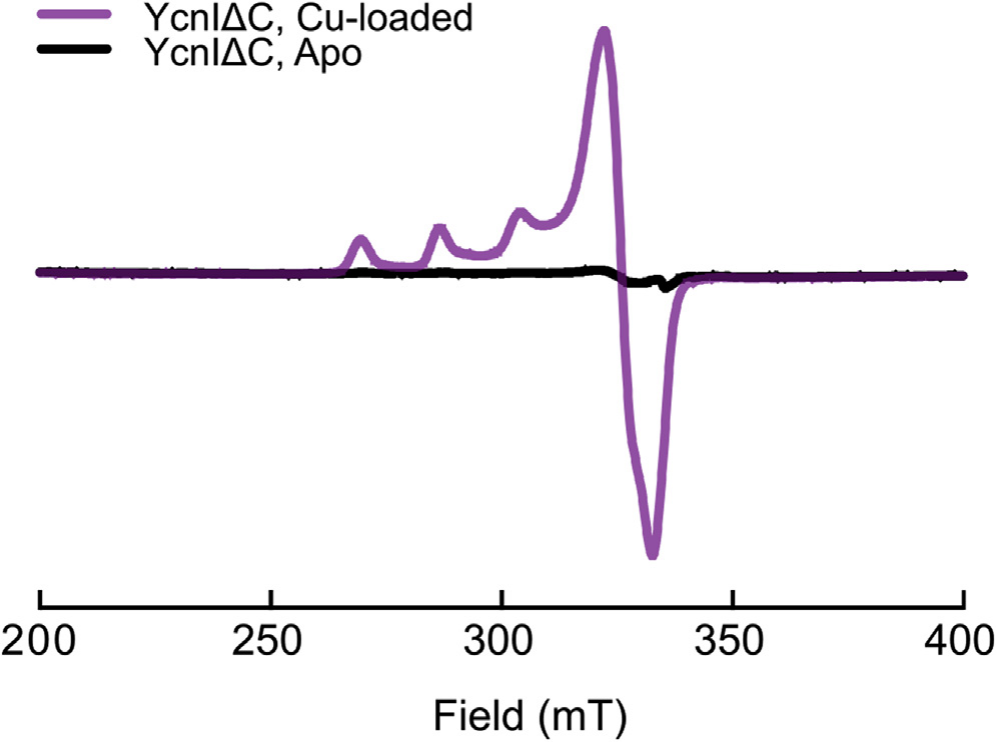
***Bs*-YcnI binds Cu(II).** EPR spectra of Cu-bound *Bs*YcnIΔC (*purple*) and apo *Bs*YcnIΔC (*black*). Instrument settings: temperature 10.0 ± 0.1 K; microwave power, 0.47 mW; modulation amplitude, 0.5 mT; modulation frequency, 100 kHz; conversion time, 88 ms; 2048 points, 16 scans. EPR, electron paramagnetic resonance.

To further investigate the copper coordination environment and determine the oxidation state of the metal, we performed electron paramagnetic resonance (EPR) spectroscopy on the Cu-loaded protein. The axial EPR spectrum clearly shows characteristic hyperfine splitting pattern in the g_||_ region indicative of Cu(II) (g_||_ ≈ 2.26 and A_||_ ≈ (17.3 ±0.4) mT; g_⊥_ ≈ 2.06; see *Experimental procedures* section for uncertainty analysis) (Fig. 2). The g_||_ and A_||_ values suggest that the Cu(II) coordination environment includes a total of four ligands comprised of either four nitrogens or two nitrogens and two oxygens (34). Consistent with the ICP-MS data, the apo protein exhibited a weak Cu(II) EPR signal indicating the presence of a small amount of bound copper. Together, these data show that *Bs*YcnIΔC binds Cu(II) in a 1:1 stoichiometry.

### *Bs*YcnIΔC adopts a cupredoxin fold and binds copper at an N-terminal site

**Figure 3:**
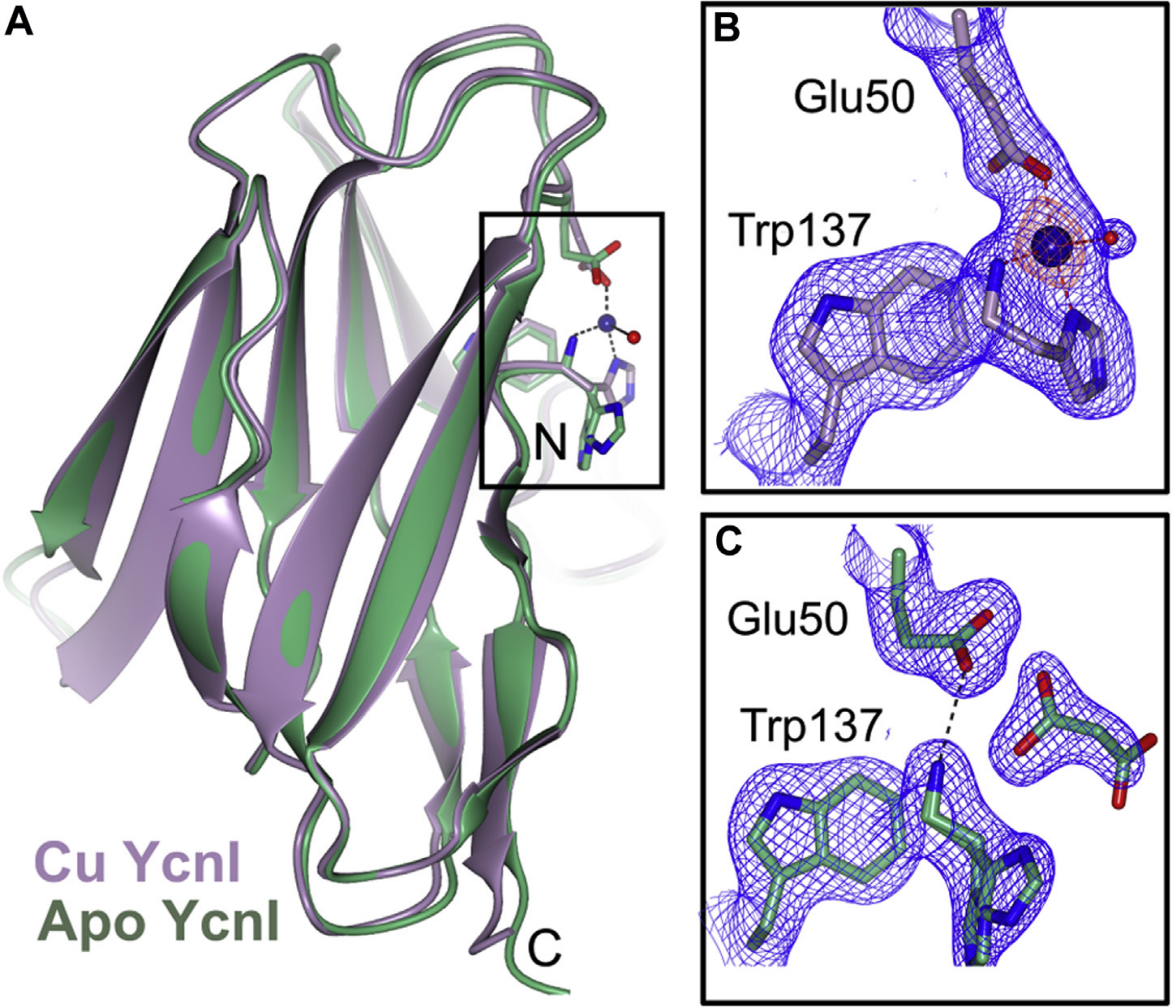
**Crystal structures of Cu-bound and apo *Bs*-YcnIΔC.***A*, superposition of the Cu-bound *Bs*YcnIΔC (*purple*) and the apo *Bs*YcnIΔC (*green*). *B*, close-up view of boxed region in the Cu-bound structure. The Cu ion is shown as a *blue sphere*. The 2*F*_o_*–F*_c_ map is shown in *blue mesh* (contoured to 1.5σ) and the anomalous density map in *orange mesh* (contoured to 5σ). Bonds are indicated by *dashed lines*. *C*, close-up view of boxed region in the apo structure, including the malonate ion in *gray*. The 2*F*_o_*–F*_c_ map is shown in *blue mesh* (contoured to 1.5σ).

We next wanted to determine precisely where the Cu(II)-binding site is in the protein as well as whether interactions with the metal induce any conformational changes, so we initiated structural studies of *Bs*YcnIΔC. We crystallized both the apo and copper-bound forms of the protein and determined their structures to a resolution of 2.05 and 2.11 Å, respectively (Fig. 3 and Table 2). Overall, *Bs*YcnIΔC adopts a cupredoxin fold featuring the characteristic Greek key β barrel shared among many bacterial copper-binding proteins (35).

**Table 2:** Crystallographic data collection and refinement statistics

Crystal	Cu *Bs*-YcnIΔC	Apo *Bs*-YcnIΔC
Protein Data Bank accession code	7MEK	7ME6
Data collection		
Wavelength (Å)^a^	1.377	0.979
Space group	*P*6_3_22	*P*6_3_22
Cell dimensions		
a, b, c (Å)	90.2, 90.2, 209.1	90.4, 90.4, 208.4
α, β, γ (°)	90, 90, 120	90, 90, 120
Resolution (Å)	30–2.11 (2.16–2.11)^b^	39.1–2.05 (2.11–2.05)^b^
*R*_meas_ (%)	13.8 (152.7)^b^	13.8 (134.5)^b^
*I/σI*	9.64 (2.64)^b^	12.06 (1.77)^b^
Completeness (%)	99.6 (98.0)^b^	99.8 (97.4)^b^
Redundancy	5.3 (5.3)^b^	11.1 (8.1)^b^
Refinement		
Resolution (Å)	29.52–2.11 (2.14–2.11)^b^	38.48–2.05 (2.10–2.05)^b^
No. of reflections	54,321	32,328
*R*_work_/*R*_free_ (%)	19.5/21.7 (30.8/30.9)^b^	19.6/21.9 (26.3/29.3)^b^
Residue range built	A: 27–155, B: 27–153	A: 27–155, B: 27–154
No. of atoms		
Protein	2027	2040
Ligand/ion	2 Cu	2 malonate
Water	125	252
Model quality		
*B*-factors (Å^2^)		
Overall	47.05	48.16
Protein	46.84	47.72
Ligand/ion	54.78	71.61
Water	50.35	50.36
RMSD, bond lengths (Å)	0.002	0.007
RMSD, bond angles (°)	0.526	0.98
Ramachandran favored/allowed/outliers (%)	97.33/2.78/0	98.81/1.19/0

a 1 Å = 0.1 nm.

b Values shown in parentheses are for the highest resolution shell.

In the copper-bound structure, we observed a strong peak in the anomalous difference map near the N terminus of each of the two molecules in the asymmetric unit that we modeled as Cu (Figs. 3*B* and S2*A*). The metal ion is coordinated both by the amino-terminal nitrogen and the δ-nitrogen of His27 at a distance of 2.0 Å, as well as the ε_2_-oxygen of Glu50 at a distance of 2.1 Å. The ε_1_-oxygen of Glu50 and, in one chain of the ASU, an ordered water molecule, coordinate more weakly from distances of 3.0 and 3.5 Å away, respectively (Fig. 3*B*). These ligands correlate well with the 2N2O coordination suggested by the EPR data, with the two nitrogen ligands deriving from His27 and the two oxygen ligands from Glu50 and a water. In addition, one of the highly conserved tryptophan residues (Trp137) further cushions the metal ion by engaging in cation–π interactions from a distance of 3.4 Å away (Fig. S2*B*). The fact that the N-terminal histidine is highly conserved across DUF1775 domains (Fig. S4) further supports the idea that it plays a vital role in binding to copper.

The fold of the apo structure of *Bs*YcnIΔC is very similar to that of the Cu-bound protein with an RMSD of 0.59 Å over 127 C_α_ (Fig. 3*A*). The primary differences are in the N-terminal region near the Cu-binding site. Instead of the His27 and Glu50 side chains turning in toward one another as they do in the Cu-bound form, in the apo form, these residues are oriented away from one another with the N terminus directly hydrogen bonding to the side chain of Glu50 (Fig. 3, *B* and *C*). In one of the two chains of the asymmetric unit, we observe two alternate conformations for His27, suggesting that this side chain displays a degree of flexibility. In our apo structure, we also observe a malonate molecule from the cryoprotectant solution near the N terminus (Fig. 3*C*), indicating that this region of the protein is solvent accessible.

### *Bs*YcnI employs a monohistidine brace motif to bind copper

**Figure 4:**
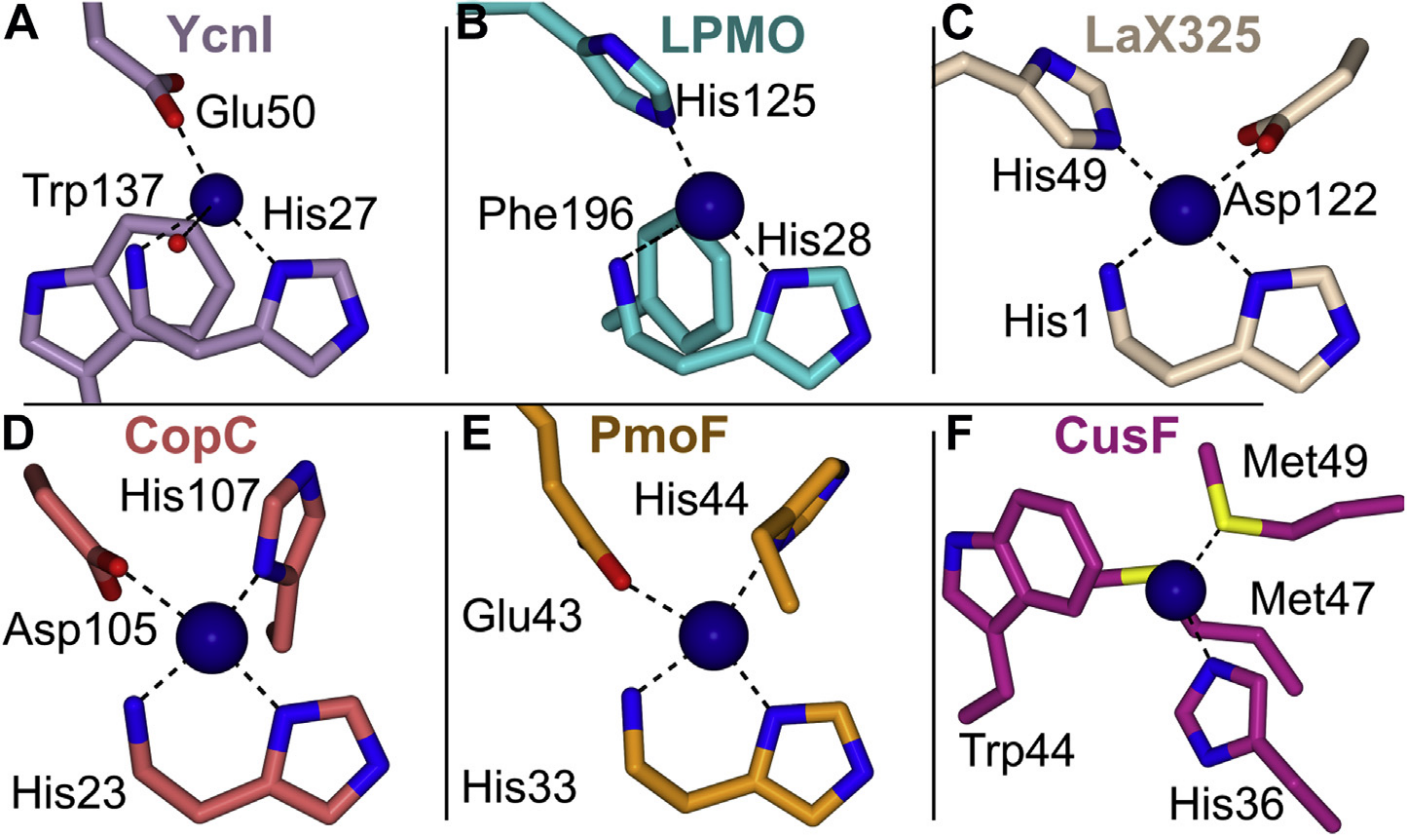
**Comparison of *Bs*YcnI with other Cu-binding proteins.** The Cu-binding sites of (*A*) *Bacillus subtilis* YcnI (this study), (*B*) *Bacillus amyloliquefaciens* CBM33 LPMO (Protein Data Bank [PDB] ID: 2YOX), (*C*) the LaX325 enzyme (PDB ID: 6IBH, chain A), (*D*) *Methylosinus trichosporium* OB3b CopC (PDB ID: 5ICU), (*E*) *Methylocystis sp.* Rockwell PmoF1 (PDB ID: 6P16), and (*F*) *Escherichia coli* CusF (PDB ID: 2VB2) shown as sticks with bonds indicated by *dashed lines* and coordinating residues highlighted. LPMO, lytic polysaccharide monooxygenase.

The *Bs*YcnIΔC crystal structure reveals a unique metal-binding site, with features reminiscent of two previously characterized Cu-binding sites (Fig. 4*A*). Most strikingly, the copper coordination observed in the crystal structure of *Bs*YcnIΔC most closely resembles the His-brace motif. The His-brace motif was initially identified in LPMOs, copper-binding enzymes that oxidatively degrade cellulose and chitin (36). In LPMOs, the His-brace motif often serves a catalytic role. Although there are differences among the active sites, LPMOs typically use a 3N T-shaped configuration and occasionally feature a phenylalanine near the active site (36) (Fig. 4*B*). Recently, an LPMO protein from *Laetisaria arvalis* (LaX325) was found to employ a His brace for copper binding as well (Fig. 4*C*), despite an absence of catalytic activity (37). Beyond LPMOs, other nonenzymatic copper-binding proteins including many members of the CopC family (Fig. 4*D*) of proteins involved in copper homeostasis (18, 32) and the PCu_A_C domains of the PmoF proteins encoded by some species of methanotrophic bacteria (17) (Fig. 4*E*) also use histidine braces to coordinate Cu ions. The His-brace motifs in all these proteins use an N-terminal histidine residue to provide two coordinating ligands to the metal: the amino terminus of the protein and one of the imidazole nitrogens of the histidine side chain (21). A second histidine residue provides a third nitrogen ligand, whereas often one or more oxygen ligands are further contributed by glutamate, aspartate, or water molecules.

Despite the marked similarities between the *Bs*YcnI Cu site and those of structurally characterized proteins that use a canonical His-brace motif, the second coordinating histidine residue is notably absent in *Bs*YcnI (Fig. 4*A*). The copper coordination site in *Bs*YcnI differs from these other proteins in that a glutamate residue completes the coordination sphere in lieu of a second histidine. Although the use of an N-terminal histidine residue as a metal ligand is a common feature among His-brace proteins, to our knowledge, all other such sites include the second coordinating histidine to form the T-shaped geometry. Our discovery of the coordination site in *Bs*YcnI introduces a new subcategory within His-brace motifs. We term this motif the monohistidine brace to differentiate it from the bis-histidine brace observed in other copper-binding proteins and enzymes. The canonical histidine brace proteins bind Cu(II) with strong affinity, from a reported *K*_*D*_ of 6 nmol l^−1^ to 31 nmol l^−1^ for several LPMO enzymes (38, 39, 40) to subfemtomolar affinity for CopC from *Pseudomonas fluorescens* (41). To determine whether the absence of a second histidine in YcnI results in a comparably reduced affinity for Cu(II) ions relative to those measured for canonical histidine brace proteins, we characterized the binding by isothermal titration calorimetry (ITC). Similar to the LPMO enzymes, we find that the binding affinity (*K*_*D*_) of *Bs*YcnIΔC for copper is ≈2 nmol l^−1^ (Fig. S3).

A second unique feature of the *Bs*YcnI copper-binding site is an adjacent tryptophan residue, Trp137, that is exceptionally well conserved across YcnI homologs. Although tryptophan residues do not traditionally engage in interactions with copper ions, there are a few notable exceptions. The CusF copper chaperone uses a somewhat similar ligand geometry (Fig. 4*F*) (42), and in the MopE protein, an oxidized tryptophan residue serves as a metal ligand (43). A recent study of CopG, a copper-dependent oxidoreductase, also features a strictly conserved tryptophan residue in close proximity to one of the metal ions (44). The orientation of the tryptophan in YcnI is most similar to that of the tryptophan in the Cu-binding site of CusF. Unlike the hydrophobic environment of the CusF tryptophan, however, Trp137 of *Bs*YcnI is located in a solvent-exposed region of the protein instead (45). Overall, the metal coordination site of *B. subtilis* YcnI appears to meld features of both the His-brace and the CusF-style copper centers using a monohistidine brace motif.

### Copper-binding residues are highly conserved in YcnI sequences

**Figure 5:**
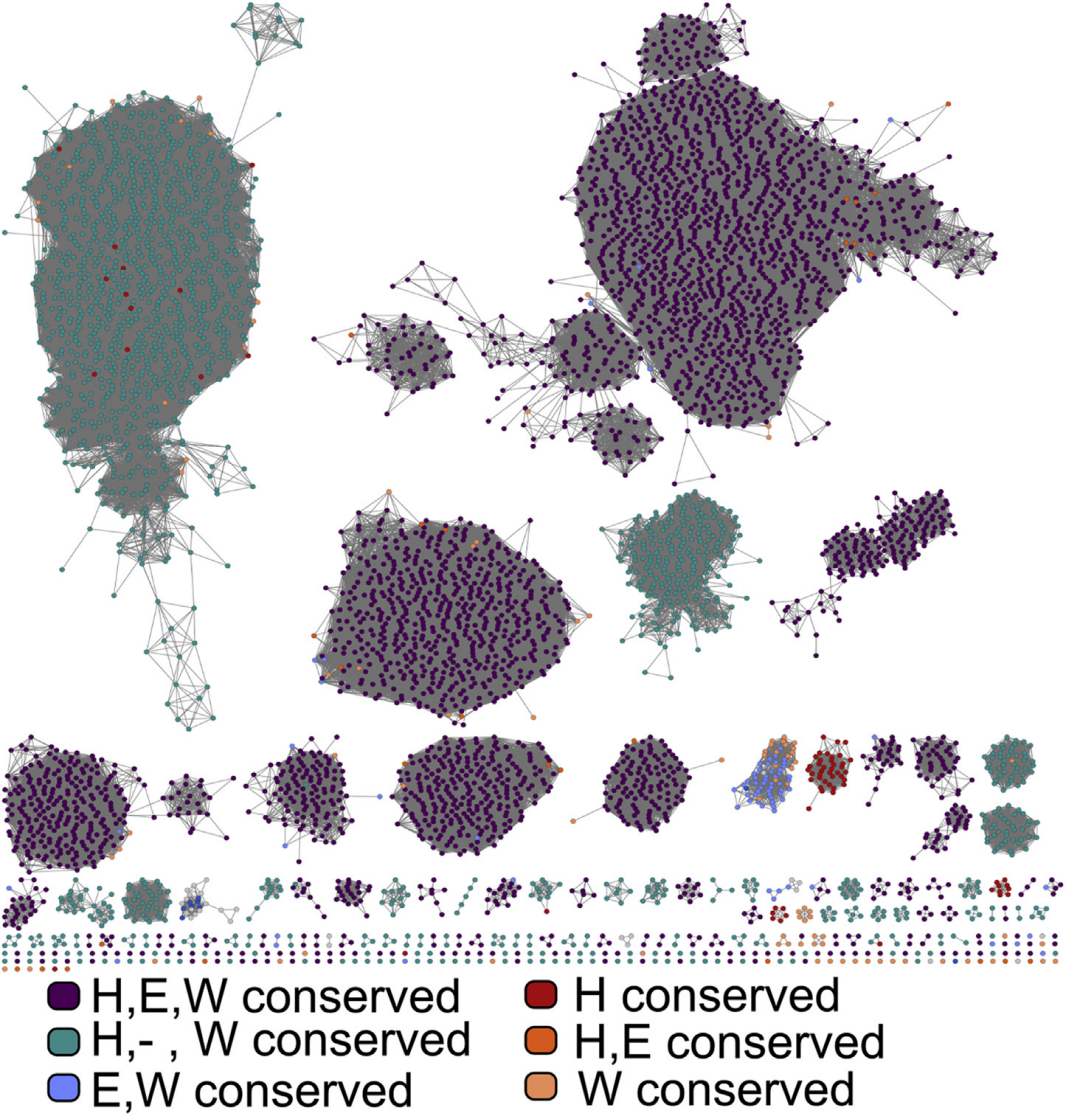
**Sequence conservation in the DUF1775 family.** Sequence similarity network for the DUF1775 family, colored by conservation of the Cu-binding ligands (His27, Glu50, and Trp137) identified in *Bs*YcnI as generated through sequence alignment in ClustalO. Sequences with all three residues conserved are indicated as *purple circles*, those with only the His and Trp conserved in *teal*, those with only the Glu and Trp conserved in *light blue*, those with the His and Glu conserved in *dark orange*, those with only the Trp conserved in *light orange*, and those with only the His conserved in *red*. DUF1775, Domain of Unknown Function 1775.

Although *Bs*YcnI and *Bradyrhizobium* *japonicum* PcuD have been implicated in copper homeostasis (8, 13), a specific role for copper binding for other YcnI family members has not been investigated directly. To gain insights into whether other homologs could engage in copper interactions in a similar fashion to *Bs*YcnI, we revisited our bioinformatics analyses to determine whether the residues we identified as the monohistidine brace motif are conserved among other DUF1775 family members as well. We aligned all sequences to the DUF1775 hidden Markov model (HMM) and found that the His, Glu, and Trp residues are conserved in most of these proteins (≈60%) (Figs. 5 and S3). Of the remaining sequences, which predominantly are found in proteobacterial genomes, the majority have the His and Trp conserved but not the Glu, suggesting that these proteins may either have a distinct function or use a different ligand set. Overall, ≈95% of the YcnI sequences have the histidine and tryptophan ligands fully conserved. These data strongly suggest that YcnI is a new player in bacterial copper homeostasis, beyond the *B. subtilis* homolog investigated here.

## Discussion

The DUF1775 domain has been suggested to play a role in copper homeostasis or trafficking, and the *Bs*YcnI protein specifically has been proposed to serve in such a capacity. Corroborating this idea, here we find that the distribution of DUF1775 domains frequently occurs among a significant number of bacterial species. Our spectroscopic data on the *B. subtilis* YcnI show that this domain binds a single Cu(II) ion, which crystallographic data further reveal to be coordinated by a solvent-accessible and N-terminal metal ion–binding site. The specific ligands that coordinate the copper ion are highly conserved among a majority of members of the DUF1775 family. Together, these data strongly suggest that YcnI, and more broadly the DUF1775 domain, represent a new class of bacterial copper-binding proteins.

*Bs*YcnIΔC binds to Cu(II) through the backbone nitrogen at the N terminus, a nitrogen from the N-terminal histidine, an oxygen of a glutamate, and a water molecule. This mono-histidine brace coordination motif and the presence of a nearby tryptophan residue represent a distinct variation on the canonical bis-histidine brace motif, raising a number of possibilities for its biological role. LPMO enzymes that use a mononuclear copper site coordinated by a bishistidine brace motif frequently exhibit oxidative properties. In these enzymes, the bis-histidine brace motif has been proposed to assist in the formation of stable and high-valent intermediates through deprotonation of the amino group (28). Studies investigating the copper-dependent monooxygenases have established a possible link between catalytic function and the bis-histidine brace (28, 46), although the CopC and PmoF proteins that also use the canonical histidine brace do not appear to have enzymatic activity (17, 18). The mono-histidine brace motif we have identified in *Bs*YcnIΔC, with its relatively low coordination number and a more solvent-exposed copper site, raises the possibility that *Bs*YcnI may not use copper for catalysis and could potentially facilitate transfer of copper ions between proteins.

Our discovery of the mono-histidine brace copper coordination site represents an important step toward understanding *Bs*YcnI, but the specific biological function of this protein remains to be fully elucidated. Since Δ*ycnJ* demonstrates a growth-defective phenotype in the absence of copper and is predicted to have a membrane-bound domain, it has been suggested to play a role in copper homeostasis (8). One possibility is that *Bs*YcnI could act as a copper chaperone to the putative copper-importing protein YcnJ. It may form a complex of high coordination number with the extracellular CopC domain of YcnJ, which in turn could facilitate copper uptake *via* the CopD domain, a putative copper importer (9). Alternately, as YcnI appears to have weaker affinity for Cu(II) than the subfemtomolar affinity reported for the CopC protein from *P. fluorescens* (41), it is possible that a reduced metal affinity of the mono-histidine brace site of YcnI compared with the bidentate site in the CopC domain could potentially promote unidirectional transfer of copper ions to the importer.

Our bioinformatics analyses indicate that YcnI homologs are found in a wide variety of bacterial species, including a number of pathogenic strains like *Mycobacterium tuberculosis*, *Listeria monocytogenes*, *Bacillus anthracis*, and others. Many of these and other homologs also share the copper-binding residues we identified in *Bs*YcnI. The existence of a subset of proteobacterial DUF1775 domains that are fused to PCu_A_C domains is also particularly intriguing in light of our findings that YcnI binds copper. It is possible that in such fusion proteins, the DUF1775 and PCu_A_C domains work in concert to maintain copper homeostasis. Because modulation of intracellular copper levels in *M. tuberculosis*, for example, can be exploited by the host immune system to combat invading pathogens (2, 47), it will be of particular importance to investigate a possible role for YcnI in bacterial copper resistance mechanisms.

## Experimental procedures

Certain commercial equipment, instruments, or materials are identified in this article in order to specify the experimental procedure adequately. Such identification neither is intended to imply recommendation or endorsement by the National Institute of Standards and Technology nor is intended to imply that the materials or equipment identified are necessarily the best available for the purpose.

### Bioinformatics analyses

To identify protein sequences that include DUF1775 domains, the IMG-JGI database was interrogated for genes encoding its corresponding PFAM (pfam07987) in finished bacterial genomes, resulting in the identification of 10,646 sequences. To generate a sequence similarity network, these sequences were submitted to the EFI-EST server using Option C. Sequences with fewer than 100 amino acids were excluded from the analysis, and an alignment score of 50 and E-value cutoff of 5 were used to generate the network. Because of the large size, for all subsequent analyses, the 100% network (in which identical sequences are represented as a single node) was used. Metadata for each sequence including pfam identifiers and taxonomic information were extracted from JGI-IMG and imported into the network table in Cytoscape (https://cytoscape.org/). A fasta file containing all sequences in the network was aligned to the HMM for PFAM07987 using ClustalO to determine the conservation of the metal-binding ligands. The identity of the amino acids at each of the positions corresponding to the metal-binding ligands was added to the metadata table (File S1). To identify gene neighbors of the aforementioned DUF1775 sequences, all genes within one position from each gene identified previously were extracted from JGI-IMG (23). These data were then used to calculate the frequency at which different domains are found in the neighboring genes to the YcnI sequence. A fasta file for the sequences in the cluster containing YcnI was generated, aligned to the HMM using ClustalO (48, 49), and the resulting file was used to generate a sequence logo in SkyLign (https://skylign.org/) (50). All software used for bioinformatics analyses are open source.

### Construct design

DNA for the soluble domain (residues 27–155) of *B. subtilis ycnI* (Uniprot ID: P94431) with a stop codon after residue 155 was synthesized into the pET28a+TEV vector using the NdeI and BamHI restriction sites. To generate the authentic N-terminal histidine residue, a His-SUMO tag was inserted immediately upstream. Briefly, the pET 28a+TEV vector was linearized using Primer Set 1 and the His-SUMO sequence was amplified using Primer Set 2 to generate matching overhangs (Table S1). The resulting DNA was assembled using Gibson Assembly to generate the His-SUMO-YcnIΔC plasmid.

### Protein expression and purification

The His-SUMO-YcnIΔC construct was transformed into BL21(DE3) cells. Overnight cultures were inoculated into Luria–Bertani media, and protein expression was induced by the addition of 1 mmol/l isopropyl β-d-1-thiogalactopyranoside at an absorbance of ≈0.6 at 600 nm. The cultures were then grown overnight at 20 °C and harvested by centrifugation at 737.5 rad/s (6000*g*) for 20 min. The pellet was resuspended in lysis buffer (150 mmol/l NaCl, 20 mmol/l 4-(2-hydroxyethyl)-1-piperazineethanesulfonic acid [Hepes], 20 mmol/l imidazole, pH 7.5) supplemented with 1 mmol/l DTT and 0.5 mmol/l PMSF prior to sonication. After sonication, the sample was centrifuged for 45 min at 1257.8 rad/s (22,036*g*). The resulting clarified lysate was applied to nickel–nitrilotriacetic acid (Ni–NTA) resin. The Ni–NTA column was then washed with three column volume lysis buffer, and His-SUMO-YcnIΔC was eluted in 150 mmol/l NaCl, 20 mmol/l Hepes, 250 mmol/l imidazole, pH 7.5. To cleave the His-SUMO tag, the eluate was incubated with His-Ulp1 protease in a lysis buffer overnight with nutation at 4 °C or dialyzed against 150 mmol/l NaCl, 20 mmol/l Hepes, pH 7.5. The untagged YcnIΔC protein was further purified by applying the cleavage products to Ni–NTA resin and collecting the flow through, which was then concentrated in 10 kDa molecular weight cutoff centrifugal concentrators (Pall). A final step of purification was performed using size-exclusion chromatography in a buffer comprised of 150 mmol/l NaCl, 20 mmol/l Hepes, pH 7.5. Peak fractions were pooled and concentrated using centrifugal concentrators. Protein concentration was measured either by an absorbance at 280 nm using an extinction coefficient of 34,950 l mol^−1^ cm^−1^ or by the Bradford assay.

### ICP-MS

Copper loading was carried out by incubating purified apo *Bs*YcnIΔC with 1 mol equivalent to 2 mol equivalents of CuSO_4_ on ice for 2 h prior to desalting using a desalting column. The concentrations of the desalted proteins were measured by absorbance at 280 nm. Both the copper-loaded samples and the apo protein samples were prepared for ICP-MS by diluting them to 0.1 μmol/l to 0.2 μmol/l in 0.239 mol/l (1% v/v) nitric acid. The ICP-MS was optimized for abundance sensitivity under hot plasma conditions with a 1 ml/min glass microconcentric nebulizer. Copper quantification followed measurement of ^65^Cu in pulse counting mode over five passes and three runs with a dwell time of 100 ms. The instrument calibration spanned less than two orders of dynamic range with five matrix-matched standards bracketing the measured copper concentrations. Calibration standards from two separate vendors were diluted from primary standards and verified against each other within the same run. Repeated analysis of unknown samples agreed within ≈2% and replicate experiments within ≈9%. All quoted uncertainties are one standard deviation statistical uncertainties from multiple repeat measurements, unless noted otherwise.

### EPR spectroscopy

Purified *Bs*YcnIΔC was incubated with 1 mol equivalent of CuSO_4_ on ice for 2 h. The sample was then desalted into 150 mmol/l NaCl, 20 mmol/l Hepes, pH 7.5 using a desalting column. The sample was concentrated to 150 μmol/l in a buffer composed of 150 mmol/l NaCl, 4.1 mol/l (30% v/v) glycerol, and 20 mmol/l Hepes, pH 7.5. A 150 μmol/l sample of apo protein for EPR studies was prepared in the same buffer. Samples were placed in precooled EPR tubes before being capped and frozen to 77 K in liquid nitrogen. Spectra were collected at (10.0 ± 0.1) K on a commercial spectrometer operating at 9 GHz using a liquid He flow-through cryostat for continuous wave EPR measurements. A power saturation series was collected to confirm that the experimental spectra presented here were collected under nonsaturating conditions. The *g* values and hyperfine coupling constants (*A*, in megahertz) reported in the article were determined directly from the spectra. From the spectra, *g* values were determined at the maxima, minima, and baseline-crossing points as reported by the analysis software provided by the instrument manufacturer. Based on the manufacturer-reported field (0.08 mT) and frequency (50 kHz) accuracies and the field resolution (≈0.1 mT per point, calculated from sweep width/number of points *viz.* 200 mT per 2048 points), the propagated uncertainty on the *g* values is ±0.002. Using the spectra, *A*_*‖*_ values were determined by measuring the difference in the field position (mT) of Cu hyperfine peak maxima at the lowest field and highest field position (that is visible) and dividing by two. The field positions of the peak maxima were determined both by a peak-picking algorithm provided in vendor instrument–supplied software and by taking the first derivative of the hyperfine peak region to determine the baseline crossing point of each peak. The two methods returned field values that agreed to within 0.1 mT for the lowest field hyperfine peak and to within 0.3 mT for the highest field hyperfine peak. Including the manufacturer-reported field accuracy (0.08 mT) and the field resolution of ≈0.1 mT per point (see previous), the propagated uncertainty on *A*_*‖*_ is calculated to be ±0.4 mT.

### Crystallization and structure determination of Cu-bound YcnIΔC

*Bs*YcnIΔC was concentrated to 12 mg/ml and incubated with 0.9 mol equivalents CuSO_4_ on ice for 1 h. Crystallization screens were set up at room temperature using a 1:1 protein:precipitant ratio in sitting drop trays. An initial crystallization hit was obtained in 0.1 mol/l citric acid at pH 3.5 and 2.0 mol/l ammonium sulfate. Crystallization conditions were further optimized to 0.1 mol/l citric acid and 2.17 mol/l ammonium sulfate. Crystals were cryoprotected by supplementing the drop with 3.57 mol/l (20% v/v) ethylene glycol. Data were collected at the 21-ID-D beamline at the Advanced Photon Source and processed to 2.11 Å resolution using XDS GUI in space group *P*6_3_22. The structure was solved by molecular replacement in phenix.phaser (51) using a structure of a closely related protein from *Nocardia farcinica* (Protein Data Bank ID: 3ESM) as a search model (top log-likelihood gain: 57.2; top translation-function *Z*-score: 8.65). AutoBuild (Phenix) was used to place additional residues in the initial solution, resulting in 246 residues in two chains in the asymmetric unit. An anomalous map was generated using phenix.maps (51) to determine the placement of copper ions. The structure was further improved by iterative rounds of model building and refinement in phenix.refine (51) and Coot (52), respectively. The final model consists of 128 residues in chain A and 126 residues in chain B with *R*_work_/*R*_free_ = 18.04%/20.24%. All software used for structure determinations and refinements are open source.

### Crystallization and structure determination of apo YcnIΔC

Crystallization screens with the apo protein were set up at room temperature using a 1:1 protein:precipitant ratio in sitting drop trays. An initial crystallization hit was obtained in 0.1 mol/l citric acid at pH 3.5 and 2 mol/l ammonium sulfate. The conditions were then optimized to 0.1 mol/l citric acid at pH 3.5 and 2.394 mol/l ammonium sulfate. Crystals were cryoprotected in 3 mol/l sodium malonate prior to harvesting. Data were collected at the 21-ID-F beamline at the Advanced Photon Source. The data were processed to a resolution of 2.05 Å in space group *P*6_3_22 using XDS GUI (53). The structure was solved by molecular replacement using the Cu-bound YcnI structure as a search model (top log-likelihood gain: 662.3; top translation-function *Z*-score: 38.0) with two molecules in the asymmetric unit. The structure was further refined using phenix.refine (51), and model building was carried out in Coot (52) resulting in the final structure with *R*_work_/*R*_free_ = 19.6%/21.9%. We also observed positive electron density in the *F*_o_–*F*_c_ map near the N terminus of both molecules in the asymmetric unit. We attempted to model metal ions as well as components of the crystallization condition, but all gave rise to unusually high >130 Å^2^ *B*-factors and did not result in improvements to the difference map. Modeling in a malonate ion because of the use of sodium malonate as a cryoprotectant did result in improved *B*-factors (71.61 Å^2^) and electron density maps.

### ITC

All ITC experiments were conducted using a MicroCal VP-ITC at 25 °C, and all samples were degassed prior to data collection. The sample cell was loaded with 25 μM apo YcnIΔC in ITC buffer (100 mmol/l NaCl, 10 mmol/l sodium acetate, and pH 5.0 buffer), and 250 μM CuSO_4_ in an identical buffer was loaded in the injection syringe. CuSO_4_ was titrated into the protein sample using injections of 10 μl with a time of 300 s between injections and constant stirring at 307 rpm. The resulting data were analyzed using commercial graphing software and fitted to a one-site binding model.

## Data availability

The coordinates and structure factors of the Cu-bound structure of YcnIΔC and of the apo structure of YcnIΔC have been deposited in the Protein Data Bank with accession codes 7MEK and 7ME6, respectively.

## Supporting information

This article contains supporting information.

## Conflict of interest

The authors declare that they have no conflicts of interest with the contents of this article.
