# Supplementary material for: The YcnI protein from Bacillus subtilis contains a copper-binding domain
Source: J Biol Chem. 2021 Aug 14;297(3):101078. doi: 10.1016/j.jbc.2021.101078 (PMC8424229; doi:10.1016/j.jbc.2021.101078)
Supplement: Table S1 and Tables S1–S4 [file mmc1.pdf]

Table S1. Primers used in construct design

Primer set	Forward Primer (5’ to 3’)	Reverse Primer (5’ to 3’)
1	atgtgagcgttaaaccggcggaga	gcgcgctcgccatatggcc
2	ggccatatggcgagcgcgatggggtccctgcaggac	tctccgccggtttaacgctcacatgacctccaatctgttcgcggt

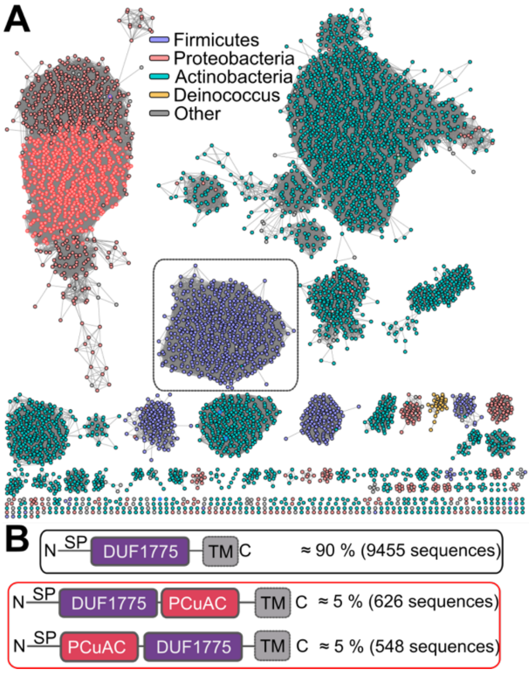

Figure S1. Bioinformatics analysis of all DUF1775 sequences. A) Sequence similarity network of 10,646 DUF1775 domain-containing protein sequences identified through JGI-IMG, colored by taxonomic information, and using an alignment score cut-off of 50. Sequences outlined in black contain only the DUF1775 domain; sequences outlined in red are fusions with PCu_AC domains. **B)** Schematics of the most common domain organizations, with outlines using same color scheme as described in A, above. SP, signal peptide; TM, transmembrane helix.

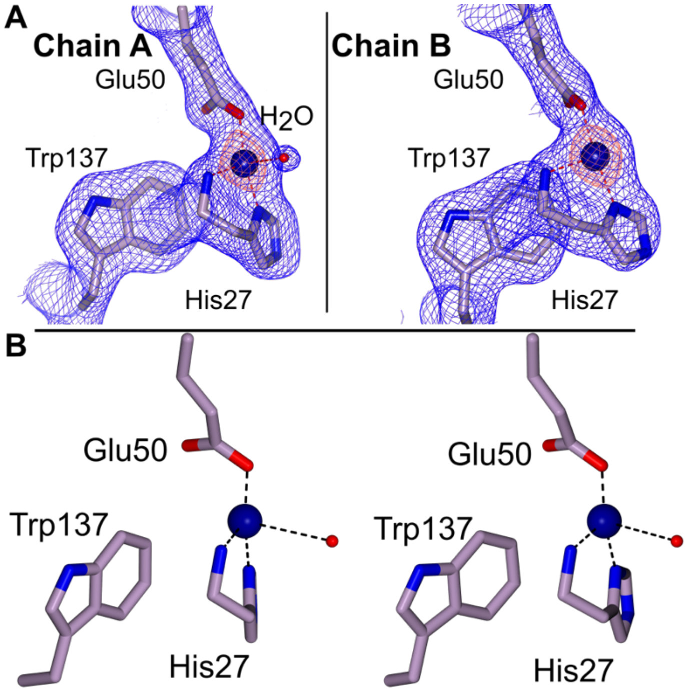

Figure S2. YcnI Cu binding site. A) Cu-binding site in Chain A (left) and Chain B (right) of the structure. $2F_o$-F_c in blue mesh, contoured at 1.5 σ; anomalous difference map in orange mesh, contoured at 5 σ. **B)** Stereoview image of the Trp137 interaction with Cu (Chain A).

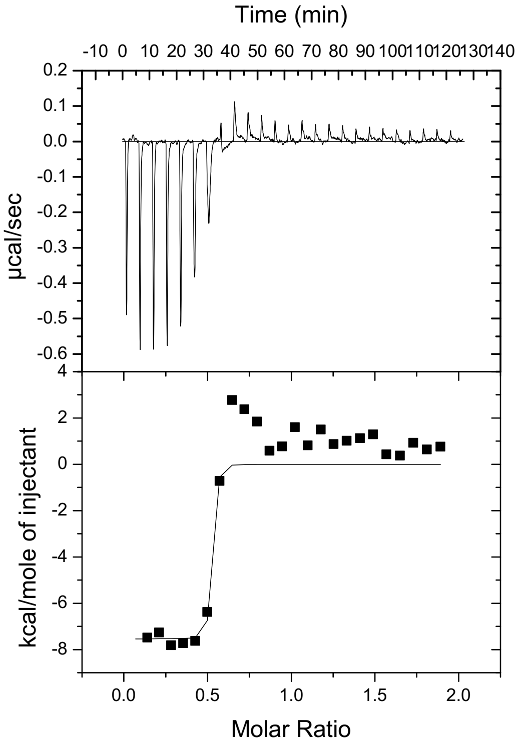

Fig. S3. Cu-binding affinity of YcnI. Representative thermogram from ITC of titration of $CuSO_4$ into YcnIΔC. Data fit to one-site binding, yielding parameters of: n = 0.497 ± 0.01; K_D = 2.4 (± 0.6) nM; ΔH = -7.54 ± 0.6 kcal/mol; ΔS = 14.1 cal/mol/K.

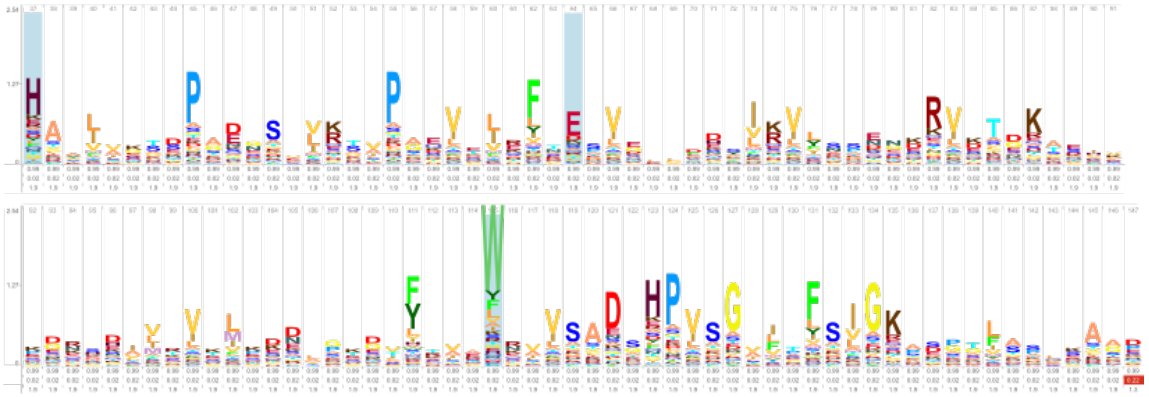

Figure S4. DUF1775 domain sequence logo. Sequence logo for all DUF1775 sequences used in the sequence similarity network. The positions of the three residues at the metal binding site (His, Glu, and Trp) are highlighted in blue.
